# An Interactive Database of Cocaine-Responsive Gene Expression

**DOI:** 10.1100/tsw.2002.145

**Published:** 2002-03-12

**Authors:** Willard M. Freeman, Kathryn E. Dougherty, Sally E. Vacca, Kent E. Vrana

**Affiliations:** Center for the Neurobiological Investigation of Drug Abuse, Department of Physiology and Pharmacology, Wake Forest University School of Medicine, Medical Center Blvd., Winston-Salem, NC 27157-1083, USA; Department of Behavioral Neuroscience and Vollum Institute, Oregon Health and Science University, 3181 SW Sam Jackson Park Road, Portland, OR 97201, USA

**Keywords:** cocaine, gene expression, microarray, protein expression, drug abuse, functional genomics, electronic publishing

## Abstract

The postgenomic era of large-scale gene expression studies is inundating drug abuse researchers and many other scientists with findings related to gene expression. This information is distributed across many different journals, and requires laborious literature searches. Here, we present an interactive database that combines existing information related to cocaine-mediated changes in gene expression in an easy-to-use format. The database is limited to statistically significant changes in mRNA or protein expression after cocaine administration. The Flash-based program is integrated into a Web page, and organizes changes in gene expression based on neuroanatomical region, general function, and gene name. Accompanying each gene is a description of the gene, links to the original publications, and a link to the appropriate OMIM (Online Mendelian Inheritance in Man) entry. The nature of this review allows for timely modifications and rapid inclusion of new publications, and should help researchers build second-generation hypotheses on the role of gene expression changes in the physiology and behavior of cocaine abuse. Furthermore, this method of organizing large volumes of scientific information can easily be adapted to assist researchers in fields outside of drug abuse.

## COCAINE ABUSE REMAINS A MAJOR SOCIETAL PROBLEM

Cocaine is a purified extract of the coca plant, Erythroxylum coca, a native of the highlands of South America. While the coca plant has been a component of the cultures of South American Indians for centuries, it was not until cocaine was purified in the mid-19$^{th}$ century that its abuse began to grow. The use of cocaine for its stimulatory and euphoric effects peaked in the 1980s with the introduction of crack cocaine – a cheaper version of the drug that can be readily smoked. Cocaine use has decreased in the U.S. from a high of almost 10 million users to around 4 million seen today, according to National Household Survey on Drug Abuse[1]. Nevertheless, the same report estimates the number of new cocaine users at over 900,000 annually, with the number of regular cocaine users remaining constant. Cocaine is the most common cause of illicit drugœrelated emergency department admissions, with over 150,000 such admissions reported annually. Clearly, cocaine abuse remains a major societal problem, and successful treatment of cocaine addiction remains difficult because recidivism rates are very high.

## INTRODUCTION

### Neurobiology of Drug Abuse

A central goal of drug abuse research is to determine the factors that contribute to the transition from recreational drug use to drug abuse and addiction. It is clear that these factors involve psychosocial, behavioral, neuroanatomical, and molecular biological components. This last facet, the molecular component, is the subject of the present discussion and this interactive database. While the social and behavioral components of cocaine abuse are substantial, growing evidence shows that long-term drug administration produces persistent changes in brain gene expression. These responsive changes in gene expression may contribute to the creation of a new neurobiological equilibrium, an allostatic state that is manifested in physiological and behavioral phenomena such as physical dependence, tolerance, withdrawal, craving, sensitization, and psychological addiction[2,3,4].

### Functional Genomics

Every cell in an organism contains the same DNA. Tissue identity and function of particular cells are therefore largely determined by the unique pattern and identities of the genes expressed within the cells of a tissue. That is, liver cells are created using one set of genes while neuronal cells use another set of genes that are unique to that cell type. The sets of genes used for creation of these different cell types may overlap but will not be identical. If a pattern of gene expression (as represented by ABC) is taken to represent a normal, or homeostatic, pattern of expression for a cell, a disease state may manifest (or be caused by) an altered, state-specific pattern of gene expression (as represented by ABC). Note that the identity of the genes remains the same, but their levels of expression have changed. This interactive database is focused on cocaine-induced changes in gene expression in the brain.

The use of animal models to identify individual genes whose expression changes in response to cocaine administration is the first step in revealing the epigenetic imprint of this drug abuse. Furthermore, gene expression changes discovered in these studies may expose important molecular targets for development of pharmacotherapeutic agents. With the completion of the human and other genome projects, traditional models of examining one gene at a time are being replaced by large-scale screening technologies. The DNA hybridization array is one such form of screening technology that permits the analysis of hundreds to thousands of genes in parallel[5]. The recent expansion of these technologies and the growth of biomedical research around the

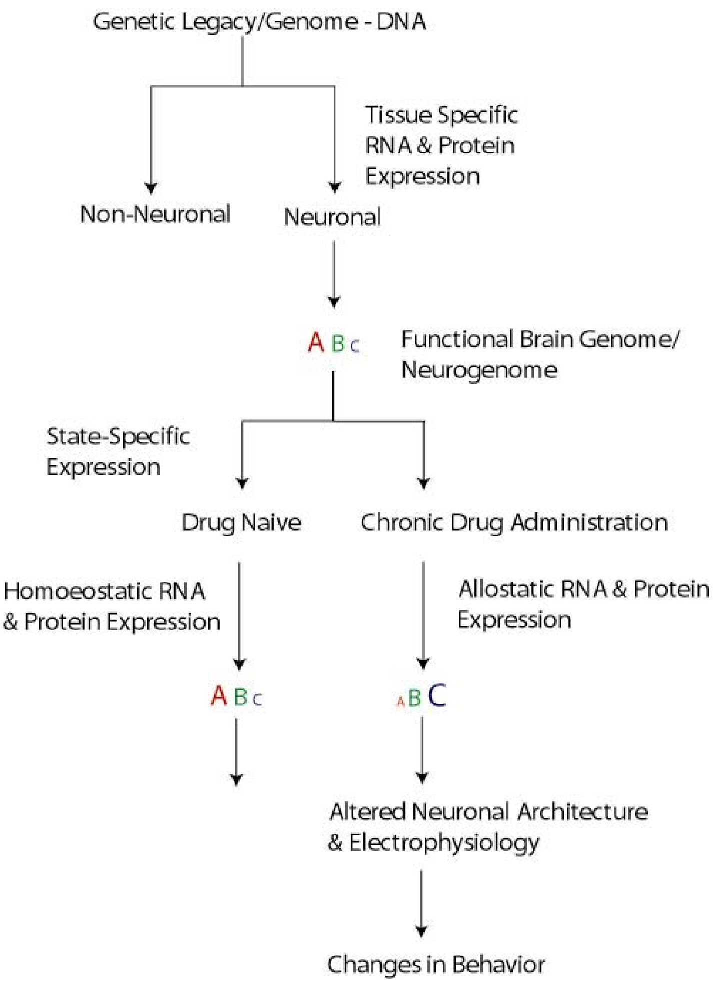

*FIGURE 1. Functional genomics and drug abuse. Chronic abuse of a drug can perturb the normal, homeostatic expression of genes. The new, allostatic pattern of gene expression may underlie the changes in behavior associated with drug abuse.*

world requires concurrent changes in traditional literature review formats. The creators of this interactive database, or dynamicREVIEW, have combined the capabilities of the Internet and interactive programming to produce a simple format that allows ready and easy access to vast quantities of information, while retaining enough detail to make the information useful.

## A NEW PUBLISHING PARADIGM: THE dynamicREVIEW

### Overview

This dynamicREVIEW is intended to provide a comprehensive, scalable, and renewable resource for information related to cocaine-induced changes in gene expression. Genes included in the database show statistically significant changes in mRNA or protein expression after cocaine treatment. Only information from whole-animal, mammalian models is included, with changes in gene expression determined by direct quantification of mRNA or protein after treatment with cocaine. Not included, although by no means unimportant, are changes observed after exposure to selective dopaminergic agents, or changes detected by methods that indirectly measure gene expression (e.g., receptor binding assays).

## Simple Organization

Cocaine-induced changes in gene expression are compiled anatomically and then placed into general functional groups. An alphabetical listing of genes is also included. Anatomical classification is necessary due to the fact that homeostatic neuronal gene expression carries a high degree of regional specificity. This review concentrates on the frontal cortex, hippocampus, nucleus accumbens, caudate/putamen (dorsal striatum), ventral tegmental area, and substantia nigra. While these are not the only brain regions affected by cocaine use, they are important components of several pathways that are critical to cocaine-related behaviors.

In addition to organizing gene expression changes by brain region, it is useful to categorize them by general function. In this interactive database, we classify genes as receptors, architectural components, secondary signal transduction factors, tertiary signal transduction proteins, biosynthetic enzymes, neuroendocrine components, transcription factors, or other gene products. These groups are intended for organizational purposes only, and are not meant to convey the precise function of every gene. While most of the groupings are self-evident, the difference between secondary signal transduction and tertiary signal transduction is intended to separate G-proteins and other small adaptor proteins from cytosolic kinases and phosphatases. As noted below, however, one advantage of the dynamicREVIEW is that the anatomical and functional grouping of genes is neither static nor final. The authors welcome editorial and/or organizational suggestions from the user.

With tens of thousands of genes in mammalian genomes, establishing the correct identity for a transcript is obviously important. To facilitate the identification of a gene, the dynamicREVIEW also provides direct links to the general genetic database OMIM (Online Mendelian Inheritance in Man), which provides instant referencing to pseudonyms and alternative nomenclatures used to identify individual genes (for example, one cocaine-induced protein, PYK2, is also known as CADTK, RAFTK, and FAK2). Furthermore, the OMIM database is routinely updated to include new data and findings concerning various genetic entities.

As currently configured, the Flash-based dynamicREVIEW provides instant access to the primary literature and is part of a Web page rather than a paper document. That is, when a specific gene product within a discrete brain region is highlighted, the original publication source can be accessed (through sciBASE and PubMed) at the click of a button. Therefore, assertions and data presented by the authors are never obscured by reviewer interpretation. This format also eliminates the need to search through articles that, despite their keywording, are not germane. Researchers can compare their results involving a particular gene with previous findings from other fields, or can look for other gene products that may interact with their gene of interest.

## A Dynamic and Living Document

As soon as a review is submitted for publication in the traditional journal environment, it becomes obsolete. Even during the review process, new information will be published. Imagine a situation in which a review is published, and some weeks later a seminal experiment is reported in the literature. With the dynamicREVIEW, the results can be added to existing reports very rapidly. Therefore, the review does not become obsolete but remains a vibrant and dynamic resource despite the passage of time. For this reason, the authors and the publisher intend to update this interactive database every 6 months. Also, unlike a paper review, the dynamicREVIEW has mechanisms in place for researchers to submit new or inadvertently omitted articles, as well as to alert the authors to errors.

## Navigating the Flash-Based dynamicREVIEW

Movement through the interactive database is simple. An opening page provides supplemental links to this text, information about the authors, and navigation instructions. The first page of the database itself shows a simplified diagram of a brain with selected regions outlined. Moving the cursor over a brain region highlights the whole region and displays its name, and clicking on the region of interest reveals a schematic of a neuron complete with the aforementioned gene groupings. Moving the cursor over the group displays the group name. When a group is selected, a list of genes is shown and individual genes may be selected. Once an individual gene has been selected, a text box and interactive links appear. The text box contains a short description of the gene and a summary of the literature on its responsiveness to cocaine. Numbered links connect to the original references. Clicking on a reference will cause the sciBASE or PubMed page for that article to appear in a new Web browser window. Moreover, there is an OMIM link, which, if selected, will spawn the appropriate OMIM entry for that gene (if available) within a new browser window. Clicking on the home button at the top right corner of every page returns the user to the first page, where he may select a new brain region. The first page of the database also includes a link to an alphabetical listing, with links, of all the genes in the database.

## Summary Interactive dynamicREVIEW Benefits

This review of cocaine-induced changes in gene expression provides instantaneous access to the primary literature underpinning the field, as well as up-to-date information regarding the new functions that are continuously being illuminated for previously characterized genes. In fact, the substance abuse field itself contributes to this phenomenon when well-studied molecular entities like PKA, PKC, or cdk are found to be cocaine responsive. The success of this new publishing environment requires that it remain a dynamic entity. Some changes will occur automatically, such as the updating of OMIM entries. On the other hand, researchers in the field are strongly encouraged to make editorial comments and bibliographic contributions, as direct submission of recent findings will permit timely updates of the review and ensure complete coverage of the field. In a similar vein, errors of interpretation or omission of pertinent findings can be readily corrected. This dynamicREVIEW will be an enduring and comprehensive tool in the investigation of epigenetic imprinting and its involvement in the long-term consequences of cocaine abuse and addiction. It is our hope that the dynamicREVIEW format will eventually be employed to assist researchers in fields beyond drug abuse.

## ACKNOWLEDGMENTS

This study was supported by U.S. Public Health Service grants P50DA06643 and R01DA13770 (K.E.V.), and T32DA07246 and T32DA07262 (W.M.F.). The authors express their gratitude to Melinda Ramsey for editorial assistance, and to Chris Richardson and Jamie McKee (at TheScientificWorld and ScienceWise, respectively) for providing the vision and assistance to make this project possible.

## BIOSKETCH

**Kent E. Vrana,** Ph.D., is a professor and director of graduate studies in the Department of Physiology and Pharmacology at the Wake Forest University School of Medicine. Dr. Vrana received a B.S. (with honors in biochemistry) from the University of Iowa and a Ph.D. in biochemistry from the Louisiana State University Health Sciences Center in New Orleans. Following a postdoctoral fellowship (in embryology and molecular biology) at the Carnegie Institution of Washington, D.C., he held a faculty position in biochemistry at the West Virginia University School of Medicine. Since 1991, he has been at Wake Forest, where he directs the Laboratory of Molecular Neurobiology. His research efforts focus on the molecular biology of the central nervous system, specifically the interactions of alcohol and drugs of abuse with genetic systems, and on biogenic amine biosynthesis. He is the author of a biochemistry textbook and more than 75 articles for biomedical books and journals. More detailed information can be obtained from his laboratory Web site (http://www.wfubmc.edu/physpharm/faculty/kev/vranak.html).

**Willard M. Freeman,** Ph.D., was a National Institute on Drug Abuse predoctoral fellow in the Department of Physiology and Pharmacology at the Wake Forest University School of Medicine. He received his doctorate in pharmacology in 2001. Dr. Freeman received a B.A. from Wake Forest University with majors in chemistry and English. Before starting graduate work at Wake Forest, he served as the laboratory manager for the Istanbul Lipid Laboratory branch of the Gladstone Institutes at the Vehbi Koç Vakfi Amerikan Hastanesi in Istanbul, Turkey. In 1999, he was a public policy intern at the National Academy of Sciences‘ Office on Public Understanding of Science. Currently, he is a postdoctoral fellow at Oregon Health and Science University in the Vollum Institute and Department of Behavioral Neuroscience. His work is centered on changes in CNS gene expression with monoaminergic drugs.

**Sally E. Vacca** is a freelance graphic artist. Ms. Vacca received her B.A. in art from Wake Forest University in 1996 and went on to study at the Portfolio Center in Atlanta, GA. Her work covers the broad range of illustration, computer animation, and sculpture.

**Katheryn E. Dougherty** is an undergraduate studying biology at Wake Forest University.
